# Bronchogenic cyst of pancreas: a rare case report

**DOI:** 10.3389/fsurg.2026.1696975

**Published:** 2026-02-19

**Authors:** Ruiqi Zou, Yushi Dai, Fuyu Li, Fei Liu, Yixin Lin

**Affiliations:** 1 Division of Biliary Tract Surgery, Department of General Surgery, West China Hospital, Sichuan University, Chengdu, Sichuan, China; 2 Department of Ultrasound, West China Hospital, Sichuan University, Chengdu, Sichuan, China

**Keywords:** bronchogenic cyst (BC), case report, pancreas, prognosis, surgical resection

## Abstract

**Background:**

Bronchogenic cyst is a rare congenital developmental abnormality of the anterior bowel. Peri-pancreatic bronchogenic cyst (PBC) is extremely rare, when only a few cases reported worldwide. Diagnosis of PBC is difficult due to the lack of specific clinical features and symptoms, laboratory and imaging findings. Currently, the diagnosis of PBC mainly relies on the comprehensive judgment of multidisciplinary collaboration, combined with clinical manifestations, imaging features and pathological verification. Most bronchogenic cysts are benign and the long-term survival rate after complete resection is close to 100%.

**Case presentation:**

A 21-year-old male with a pancreatic mass was admitted to our hospital. CT scan showed a low-density cystic shadow about 3.6 × 3.5 cm^2^ on the pancreatic body, with clear boundaries, no enhancement, and no dilation of the main pancreatic duct. The patient underwent open distal pancreatectomy. Postoperative histopathological findings allowed for a definitive diagnosis of PBC. There was no recurrence for two years postoperatively.

**Conclusion:**

In this study, we reported a rare case of PBC. At present, radical surgical resection remains the most effective treatment for bronchogenic cyst with mass effects, infection, bleeding, rupture, or larger than 3 cm in diameter. Further research on the diagnosis and treatment strategies of PBC is required.

## Introduction

Bronchogenic cysts are rare congenital abnormalities of foregut development that originate from lung buds and are present at birth (1). While most commonly located in the mediastinum, these cysts can also manifest in other locations, including the diaphragm, abdominal cavity, and subcutaneous tissues (1). Among these ectopic presentations, peri-pancreatic bronchogenic cysts (PBCs) are exceptionally rare, with only a few cases documented globally (2). Although histologically benign, PBC poses significant diagnostic challenges. This difficulty stems from the absence of specific clinical symptoms, laboratory markers, or pathognomonic imaging features. Furthermore, their clinical presentation is often insidious, and their radiological appearance closely mimics that of other more common pancreatic cystic lesions, such as pseudocysts and mucinous cystadenomas. Consequently, PBCs are frequently misdiagnosed or overlooked (3, 4). Current diagnostic strategies therefore necessitate a multidisciplinary approach, relying on the integration of clinical findings, detailed imaging characteristics, and ultimately, definitive pathological confirmation (5). Endoscopic ultrasonography (EUS)-guided through-the-needle biopsy (EUS-TTNB) is a feasible method for precise histological diagnoses before operation (6, 7). It is noteworthy that, similar to mucinous cystic neoplasms, bronchogenic cysts have been reported to harbor malignant potential (8). However, the specific triggers for malignant transformation in PBC remain entirely unknown. Given its rarity and diagnostic complexity, accumulating detailed case reports describing the comprehensive clinical, radiological, and pathological features of PBC is crucial to enhance our understanding of this entity. In this context, we present a case of PBC successfully managed with open distal pancreatectomy and splenectomy, contributing to the limited literature on this condition.

## Case presentation

**Figure 1:**
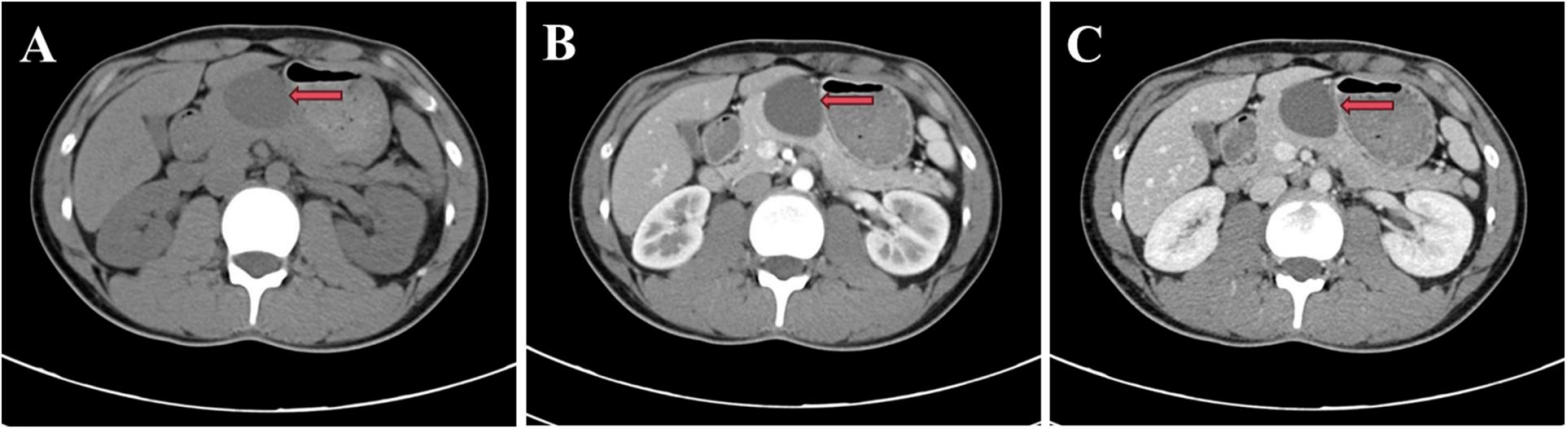
The lesion in the pancreatic body was detected on CT scan, which was marked by red arrows. (**A**, plain scan; **B**, arterial phase; **C**, venous phase).

**Figure 2:**
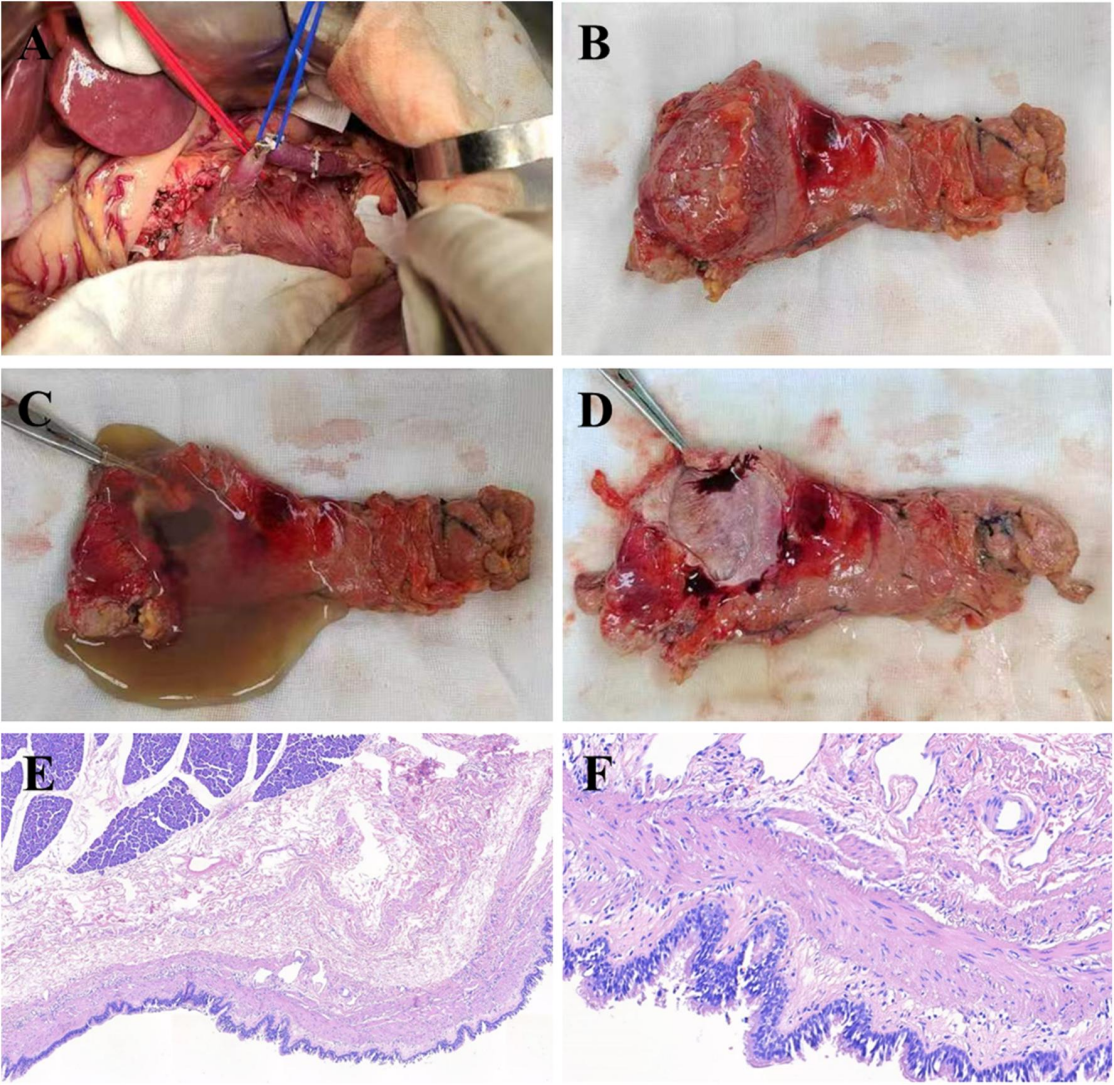
The patient underwent open distal pancreatectomy **(A)**, gross examination showed a pancreatic mass are located in the body of pancreas **(B)** after the pancreatic mass was opened, a light yellow, soft, gelatinous substance flowed out, and the cystic cavity was smooth and no new organisms were found **(C,D)**. Histopathological analysis showed that the cyst wall of the pancreatic mass was lined with a pseudo-lamellar fibrous columnar epithelium, which was considered to be a bronchogenic cyst of the pancreas **(E**. HE staining, ×100; **F**. HE staining, ×400).

A 21-year-old male presented with intermittent upper abdominal discomfort. Diagnostic evaluation revealed a pancreatic mass. The patient reported no history of industrial chemical exposure or prior radiotherapy. Physical examination was unremarkable. Laboratory investigations, including comprehensive metabolic panel, hepatic and renal function tests, and tumor markers [carcinoembryonic antigen (CEA), cancer antigen19-9 (CA19-9), carbohydrate antigen 125 (CA125)]—were within normal limits. Contrast-enhanced abdominal CT demonstrated a well-circumscribed, non-enhancing hypodense cystic lesion (3.6 × 3.5 cm^2^) in the pancreatic body without main pancreatic duct dilation (Figures 1A–C). Subsequent contrast-enhanced ultrasound corroborated these findings, identifying a 4.2 × 3.2 cm^2^ cystic structure in the same location. Based on these features, a preoperative diagnosis of pancreatic cystic lesion was established. Following multidisciplinary review, the patient underwent open distal pancreatectomy with splenectomy (Figure 2A). Intraoperative inspection identified a discrete mass within the pancreatic body (Figure 2B). Upon incision, the cyst released viscous, light-yellow gelatinous material; the cystic cavity exhibited a smooth lining without mural nodules or neoplastic growth (Figures 2C,D). Histopathological analysis confirmed the diagnosis: the cyst wall was lined by pseudostratified ciliated columnar epithelium supported by fibroconnective tissue containing submucosal glands and smooth muscle bundles—pathognomonic features of a bronchogenic cyst (Figures 2E,F).

**Figure 3:**
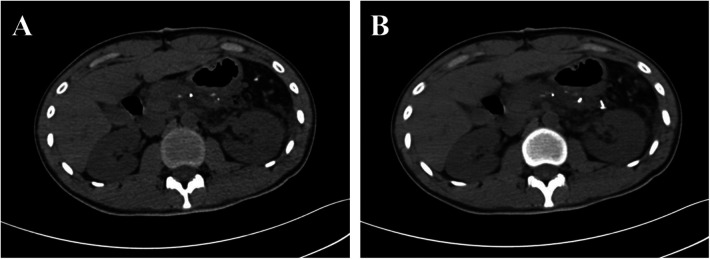
CT showed the patient was free from tumor recurrence after 2 years' following up. (**A** and **B**, Plain scan).

The patient's postoperative course was uneventful. He was discharged on postoperative day 7 with stable vital signs and normalized laboratory parameters. Surveillance protocol included regular outpatient follow-up with serial imaging. At 24-month follow-up, contrast-enhanced CT confirmed no evidence of recurrence, supporting the benign nature of this rare entity (Figures 3A,B).

## Discussion

Bronchogenic cyst is a rare congenital developmental abnormality of the anterior bowel (1), which is commonly found in the mediastinum (9) or lung (10), and occasionally in the diaphragm (11), neck (12), and abdominal cavity (13) (Table 1). Bronchogenic cysts of the pancreas are extremely rare (2). Clinical manifestations of PBCs are notably nonspecific. Most remain clinically silent unless complications develop, such as: Secondary infection leading to inflammatory symptoms; Significant cyst enlargement causing mass effect on adjacent viscera. Symptomatic presentations typically reflect mechanical compression, including nausea, vomiting, and epigastric discomfort when substantial cyst expansion impinges on neighboring structures like the stomach or duodenum.

**Table 1:** Ectopic bronchogenic cyst in different locations.

Location	Age (Year)	Sex	Symptom	Size (Maximum diameter)	Intervention	Reference
Head and Neck						
Tongue	5-month	Male	Feeding difficulty	3.0 cm	Resection	(25)
Suprasternal fossa	31	Female	Asymptomatic	2.5 cm	Resection	(26)
Peri-Thyroid	27	Female	Hoarseness	4.0 cm	Resection	(27)
Peri-Thyroid	28	Female	Asymptomatic	5.0 cm	Resection	(27)
Thorax						
Interatrial septum	42	Male	Palpitation	2.9 cm	Thoracotomy resection	(28)
Diaphragmatic	50	Male	Asymptomatic	7.0 cm	Thoracotomy resection	(29)
Posterior mediastinum	37	Male	Asymptomatic	3.7 cm	Thoracoscopic resection	(30)
Mediastinum	59	Male	Dysphagia	3.0 cm	Thoracoscopic resection	(31)
Abdomen						
Peri-Splenic	48	Female	Asymptomatic	2.6 cm	Laparoscopic resection	(32)
Peri-Stomach	21	Female	Asymptomatic	6.2 cm	Laparoscopic resection	(33)
Peri-Hepatic	Not mentioned	Male	Abdominal pain	8.0 cm	Fenestration drainage	(34)
Retroperitoneal	51	Male	Asymptomatic	5.9 cm	Laparoscopic resection	(35)
Others						
Intradural extramedullary	35	Male	Dysuria	1.3 cm	Resection	(36)
Cutaneous of back	9	Female	Exudation	Not mentioned	Biopsy	(37)

Diagnostic challenges persist due to the absence of pathognomonic features across clinical presentation, laboratory parameters, and conventional imaging. Symptom emergence often serves as the primary indicator necessitating further investigation (14). While contrast-enhanced CT and MRI provide essential morphological characterization, several factors complicate radiographic differentiation: Variable cyst fluid composition (proteinaceous content, calcium deposits); Size-dependent enhancement patterns; Secondary inflammatory changes from superinfection; Similar appearance to more common pancreatic cystic neoplasms (15, 16).

It is reported that EUS-guided through-the-needle biopsy (EUS-TTNB) facilitates definitive histological characterization prior to intervention (17). Cytological diagnosis via EUS-FNA predominantly relies on demonstrating ciliated epithelial cells within aspirated fluid samples (18). Current diagnostic paradigms necessitate multidisciplinary integration of clinical context, advanced imaging, and ultimately histopathological verification (5). However, a wrong diagnosis of nature is common in pancreatic cystic neoplasms (PCNs). About one-fifth of resected PCNs remained preoperatively misdiagnosed, despite according to international consensus guidelines, the EUS has been widespread used (19).

Surgical management remains controversial, particularly for asymptomatic cysts. Conservative observation may be considered for incidentally discovered, radiologically classic lesions. Several authorities advocate prophylactic excision given the diagnostic uncertainty and oncological concerns (20). Compelling arguments for resection include: Parallels with mucinous cystic neoplasms (10%–15% malignancy rate, size-dependent risk) (21); Established malignant potential, with documented cases of carcinomatous transformation (22, 23); Prevention of infectious, hemorrhagic, or obstructive complications; Resolution of symptomatic mass effect (23).

In the present case, preoperative evaluation could not exclude a mucinous cystic neoplasm, prompting open distal pancreatectomy. Prognostically, complete resection of benign bronchogenic cysts typically yields excellent outcomes with near 100% long-term survival (3, 24). Nevertheless, critical considerations include: Mandatory histopathological confirmation to exclude malignant elements; Documentation of cyst wall integrity and epithelial characteristics; Need for longitudinal surveillance data to establish PBC-specific malignant risk profiles.

## Conclusions

In summary, we have documented a rare case of pancreatic bronchogenic cyst (PBC). The patient underwent open distal pancreatectomy following preoperative identification of a pancreatic cystic lesion, with definitive histopathological confirmation of PBC. Current evidence indicates that complete surgical resection remains the optimal therapeutic approach for bronchogenic cysts presenting with: Significant mass effect; Infectious complications; Hemorrhagic manifestations; Rupture risk; Diameter exceeding 3 cm. Notably, radical excision generally yields excellent long-term outcomes. Further research is warranted to refine diagnostic algorithms and optimize management strategies for this rare entity.

## Data Availability

The raw data supporting the conclusions of this article will be made available by the authors, without undue reservation.
